# Iso-α-acids in Nonalcoholic and Alcoholic Beer Stimulate Growth of Neuron-like SH-SY5Y Cells and Neuroepithelial Stem Cells

**DOI:** 10.1021/acsbiomedchemau.1c00017

**Published:** 2021-09-07

**Authors:** Agneta J. Laurent, Niels Bindslev, Vladana Vukojević, Lars Terenius

**Affiliations:** † Department of Clinical Neuroscience, Center for Molecular Medicine, Karolinska Institutet, SE-171 76 Stockholm, Sweden; ‡ Department of Biomedical Sciences, Faculty of Health and Medical Sciences, University of Copenhagen, Blegdamsvej 3B, DK-2200 Copenhagen N, Denmark

**Keywords:** nonalcoholic/alcoholic beer, iso-α-acids, SH-SY5Y cells, neuroepithelial stem cells, peroxisome proliferator-activated receptor, PPAR, PPARα GW6471, PPARγ GW9662, CB1 Ibipinabant, neurogenesis

## Abstract

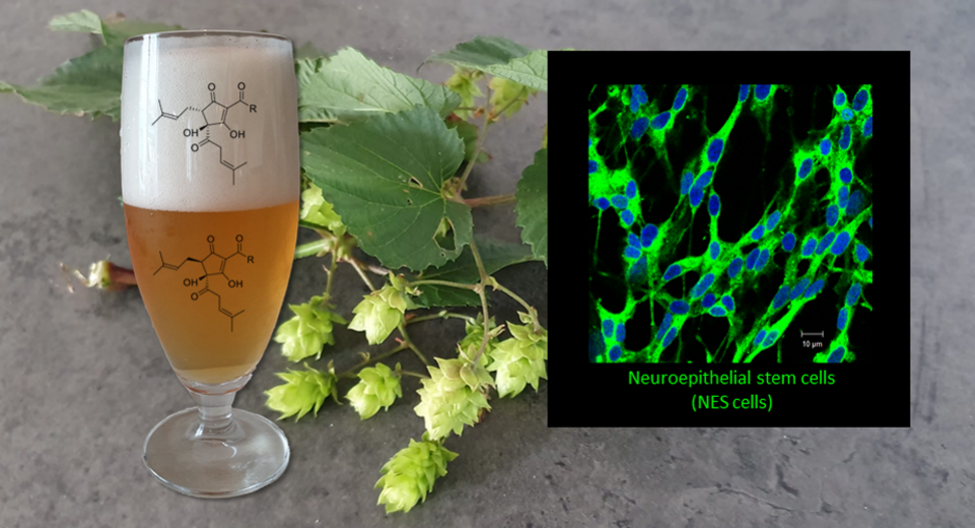

With the increasing popularity of nonalcoholic beer, the association between beer drinking and alcohol intake is lost. In the present study, we show that nonalcoholic beer can stimulate the expansion of neuron-like cell lines and neuroepithelial stem cells in culture, yielding an effect comparable to that of alcoholic beer. One ingredient in beer is hops, which is derived from the flower of hop plants. The female flower contains humulones, which are transformed into iso-α-acids during wort boiling and give beer its bitter taste. In this study, we tested the effects of these iso-α-acids and/or alcohol on the proliferation of neuron-like cells and neuroepithelial stem cells in culture. Iso-α-acids enhanced cell expansion, showing a bimodal dose–response curve with peaks around 2–30 nM and 2–5 μM, of which nanomolar concentrations are relevant in beer drinking. The more lipophilic *trans*-iso-α-acids, found to a greater extent in beer foam, are even more potent. Our results indicate that iso-α-acids, acting via peroxisome proliferator-activated receptors could be responsible for the observed effects. Altogether, our results indicate that nonalcoholic beer with ingredients such as iso-α-acids stimulate the proliferation of neuroepithelial stem cells.

## Introduction

Beer has been part of the human diet for over 7000 years and is the most consumed alcoholic beverage in the world. There are risks with drinking too much alcoholic beer, and the national recommendations of a maximum 7 standard drinks per week for women and 14 for men is now even reduced in some countries. While too much alcoholic beer can give consequences such as alcoholism and addiction, low to moderate beer intake has on the other hand been considered healthy.^1^ Whether these health effects come from the alcohol or from other ingredients in beer is, however, not completely understood, and the health effects of nonalcoholic beer compared with alcoholic beer are relatively unexplored.

During the 21st century, there has been a trend toward a healthier lifestyle, including the development and increased consumption of nonalcoholic beer.^2^ Nonalcoholic beer has increased in popularity and is now also brewed by many microbreweries. Consumers prefer it not only because it is innovative and trendy but also because of increased health awareness. Nonalcoholic beer is low in calories, is considered safe, and can even be consumed during pregnancy. It has also become an attractive alternative to alcoholic beer in countries where the use of alcohol is restricted.

**Figure 1:**
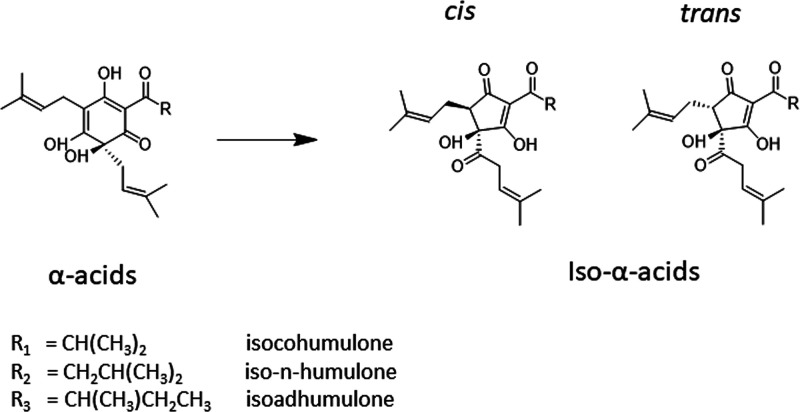
Isomerization of α-acids to *cis-* and *trans*-iso-α-acids.

Beer contains a variety of nutrients and bioactive compounds with different health-promoting effects.^1^ One of the main ingredients in beer is hops from the female flower of the hop plant, *Humulus lupulus* L. As a plant from the Cannabaceae family, hops contain many bioactive compounds.^3^ Hops have been used in brewing since the Middle Ages and in traditional medicine since ancient times.^4,5^ Among the bioactive compounds are α-acids, which are found in the glands of the flowers. During wort boiling, these α-acids are isomerized to iso-α-acids, the bitter component in beer^4^ (Figure 1).

Many neurodegenerative disorders are characterized by neuroinflammation and cell death, where age is the main risk factor. In an aging society, there is an increasing need for compounds that protect against cell death and that stimulate neurogenesis. Ingredients in beer, other than alcohol, may have preventive effects on neurodegeneration. Iso-α-acids have been shown to be neuroprotective, can penetrate into the brain,^6^ and have been shown to reduce levels of amyloid β, improve microglia function, suppress inflammation, and improve memory function.^6−9^ Whether iso-α-acids also generate neurogenesis is not well-studied.

To determine whether the neuroprotective effects of alcoholic beer are due exclusively to ethanol or are due, in part, to nonalcoholic components, we performed cell proliferation assays with neuron-like cell lines and human neuroepithelial stem (NES) cells. The effects of nonalcoholic beer, with iso-α-acids and very low levels of ethanol, were compared against alcoholic beer, with iso-α-acids at the same level. The potency of the more lipophilic *trans*-iso-α-acids, found in a greater extent in beer foam, was also studied. In addition, possible actions of *trans*-iso-α-acids via peroxisome proliferator-activated receptors (PPARs) were studied, as iso-α-acids previously have been shown to act via PPAR.^10^ It has been shown that ligands of the cannabinoid receptor CB_1_ act either directly or indirectly via PPAR.^11^ As hops belong to the Cannabacea family, possible actions via the CB_1_ receptor were also investigated.

## Materials and Methods

### Iso-α-acids

Isomerized hop extract, Hopsteiner Iso-Extract 30%, kindly provided by Hopsteiner (Mainburg, Germany), was purified at SciLifeLab, Stockholm, Sweden. Preparative high performance liquid chromatography (HPLC) was performed on a Gilson 305 HPLC system using an acidic eluating protocol. For acidic purification, the Gilson 305 HPLC system was equipped with an ACE 5 C8 (5 μm, 30 mm × 150 mm) column, and the compounds were eluted using a gradient system of acetonitrile and H_2_O containing 0.1% TFA. Analytical reversed-phase liquid chromatography–mass spectrometry (RPLC-MS) was performed using an Agilent/HP 1200 system 6110 mass spectrometer with electrospray ionization (ESI+). For the HPLC-MS method, the ACE C18 3.5 μm column (3.0 mm × 50 mm) was used. The mobile phase [0.1% TFA/CH_3_CN]/[0.1% TFA/H_2_O] and positive electrospray ionization was used for detection/characterization. The isomer fractions were combined, dried, and dissolved in 10 mM dimethyl sulfoxide (DMSO). The purity of the collected isomers were 87% (UV, 254 nm) considering peaks with a retention time between 0.7 and 3.5 min and with a height above 30 mAU (Supporting Information, Figures S1–S3). For comparison between different iso-α-acids, Thermo Scientific BeerNHop Solution (in methanol, MeOH) at a concentration of 276 μM was purchased from Thermo Fisher Scientific (Waltham, MA, USA). The initial experiments were done with iso-α-acids from Hopsteiner (purity 87%) and confirmed with Thermo Fisher (purity >95%). Both sources yielded equivalent results.

*Trans*-iso-α-acids, DCHA-Iso, ICS-I4, was purchased from Labor Veritas AG (Zürich, Switzerland) and dissolved in DMSO at a concentration of 100 mM. Isomers from different iso-α-acids are illustrated in Figure 1.

Reduced forms of iso-α-acids including *cis*-ρ-iso-α-acids, DCHA-Rho, ICS-R3; tetrahydroiso-α-acids, Tetra, ICS-T3, and *cis*-hexahydroiso-α-acids, DCHA-Hexa, ICS-H2 (Supporting Information, Figure S4), were purchased from Labor Veritas AG and dissolved in methanol or DMSO.

### Cells and Cell Cultures

The human neuroblastoma SH-SY5Y cell line and Neuro-2a (N2a) cell line from mice, both from American Type Culture Collection (Manassas, VA, USA), were cultured in nontreated flasks in Dulbecco’s modified Eagle medium (DMEM) supplemented with 10% fetal bovine serum, 100 U/mL penicillin, and 100 μg/mL streptomycin (all from Gibco, Thermo Fisher Scientific) at 37 °C in a humidified atmosphere containing 5% CO_2_. Cell culture medium was changed every second to third day. Trypsin-EDTA (Gibco) was used to dissociate adherent cells from the flask during routine cell culture passaging.

Neuroepithelial stem (NES) cells derived from the human-induced pluripotent stem (hiPS) cell line CTRL-09-II PS by directed differentiation^12,13^ were provided by Anna Falk Lab/iPS Core Facility, Karolinska Institutet, Stockholm, Sweden. The cells were cultured at a density of 4 × 10^4^ cells/cm^2^ in flasks coated with 20 μg/mL poly-l-ornithine (Sigma, St. Louis, MO, USA) and 2 μg/mL laminin 2020 (Engelbreth–Holm–Swarm murine sarcoma, Sigma) in DMEM/F12+GlutaMax (Gibco) supplemented with 1% N2 (Gibco), 0.1% B27 (Gibco), 10 ng/mL of basic fibroblast growth factor (Gibco), 10 ng/mL of human epidermal growth factor (PeproTech, Rocky Hill, NJ, USA), and 100 U/mL penicillin with 100 μg/mL streptomycin (Gibco) at 37 °C in a humidified atmosphere containing 5% CO_2_. Medium was changed every second day, and TrypLE Express (Gibco) was used for passaging.

### Proliferation and Expansion Assay

SH-SY5Y cells were cultured in nontreated 24-well plates (Sarstedt, Nümbrecht, Germany) in 500 μL of DMEM/well supplemented as indicated above. SH-SY5Y cells were seeded at a cell density of 1 × 10^4^ cells/well and cultured without treatment or with ethanol, iso-α-acids, or *trans*-iso-α-acids without/with ethanol; *cis*-ρ-iso-α-acids, *cis*-hexahydroiso-α-acids, or tetrahydroiso-α-acids for 7 days.

Carlsberg Alcohol Free (0.5% ABV, alcohol by volume) or Carlsberg Export (5.0% ABV) were used in beer-treatment experiments. Both Carlsberg Alcohol Free and Carlsberg Export are pilsner style beer containing 18 international bitterness units (IBU) or 18 mg of iso-α-acids/L (Sweden). The volume of beer was added to reach a certain EtOH or iso-α-acid concentration. In beer-treatment experiments, 3.5 × 10^4^ SH-SY5Y cells/well were plated and treated with Carlsberg Alcohol Free (0.5% ABV) or Carlsberg Export (5.0% ABV) for 4 days. N2a cells were seeded at a density of 1 × 10^4^ cells/well in nontreated 24-well culture plates (Sarstedt) in 500 μL of DMEM/well supplemented as indicated above. N2a cells were cultured without treatment or with ethanol or iso-α-acids without/with ethanol; trans-iso-α-acids or cis-ρ-iso-α-acids for 4 days.

The NES cells were cultured in 20 μg/mL poly-l-ornithine and 2 μg/mL laminin-coated 24-well culture plates (Sarstedt) in 500 μL of NES medium supplemented as indicated above. The NES cells were seeded at a density of 1.5 × 10^4^ cells/well and cultured without treatment or with ethanol, iso-α-acids purified from Hopsteiner Iso-Extract 30% (Hopsteiner) without/with ethanol or *trans*-iso-α-acids for 7 days. In beer-treatment experiments, NES cells were seeded at a density of 5 × 10^4^ cells/well and treated with Carlsberg Alcohol Free (0.5% ABV) or Carlsberg Export (5.0% ABV) for 3 days. Treatments are listed in Table S1, Supporting Information. After the initiation of treatments, the cell culture medium was not changed. In control experiments with medium only and no treatment, the cell culture medium was replaced in the same way as in the treatment experiments.

### Flow Cytometry

Cells were analyzed on a BD FACSVerse flow cytometer with BD FACSuite software (BD Biosciences, San Jose, CA, USA). Further analyses were performed using the software Flowjo version 10 (Flowjo, LLC, Ashland, OR, USA).

### Proliferation Assay with Carboxyfluorescein Succinimidyl Ester (CFSE)

SH-SY5Y cells were stained with 2 μM CFSE (Invitrogen, Carlsbad, CA, USA), incubated at 37 °C for 15 min in a humidified atmosphere containing 5% CO_2_ and washed twice in phosphate-buffered saline (Sigma) supplemented with 0.5% fetal bovine serum (Gibco). Cells stained with CFSE were then incubated as described above.

### Proliferation Assay with 5-Bromo-2′deoxyuridine (BrdU)

SH-SY5Y cells were incubated with iso-α-acids purified from Hopsteiner Iso-Extract 30% (Hopsteiner) in the range of 2.76 nM to 27.6 μM or with *trans*-iso-α-acids in the range of 2.76 nM to 27.6 μM without/with 2 mM ethanol for 48 h. The cells were incubated with BrdU-pulsing solution provided with the phase-flow BrdU cell proliferation kit (BioLegend, San Diego, CA, USA) for the last 20 h of the total incubation time of 48 h and then fixed, permeabilized, and treated with DNase (all provided in the kit), and stained with anti-BrdU antibody, 7-aminoactinomycin D (7-AAD), and fluorochrome-conjugated monoclonal antibody Brilliant Violet 421 anti-human CD56 (NCAM) clone HCD56 according to the protocol from the supplier (all provided by BioLegend).

### Evaluation of Cell Expansion and Proliferation

After completing the incubation, the cells were deattached with trypsin-EDTA (Gibco) for SH-SY5Y or N2a cells and TrypLE Express (Gibco) for NES cells, assessed for viability using Trypan blue (Sigma) and counted with a Blaubrand Bürker hemocytometer (Sigma). Cell growth was quantified as the total number of cells on day 3 (NES), 4 (SH-SY5Y, N2a, NES), or 7 (SH-SY5Y, NES) divided by the number at the start and compared with the growth in the control wells assessed in the same way. CFSE-based cell proliferation was analyzed with flow cytometry described above and quantified as the percentage of CFSE^low^ cells in treated cells compared with the percentage in control wells. The same evaluation procedure was also applied using the BrdU^+^ in the proliferation assay.

### Expansion Assay with Agonist and Antagonists

SH-SY5Y cells (1 × 10^5^ cells/cm^3^), cultured in nontreated 24-well culture plates (Sarstedt) for 48 h, were treated with *trans*-iso-α-acids in the concentration range of 276 pM to 2.76 μM without/with antagonists of PPARα, PPARγ, or cannabinoid-1 (CB_1_) receptor at twice the concentration of *trans*-iso-α-acids. The cells were deattached with trypsin-EDTA (Gibco); the number of cells per well was determined with a hemocytometer, and expansion was calculated as described above. The following three antagonists were used: PPARα antagonist GW6471 (IC_50_ 0.24 μM) and PPARγ antagonist GW9662 (IC_50_ 3.3 nM) (both from Sigma) and CB_1_ receptor antagonist Ibipinabant (IC_50_ 22 nM; Medchemtronica, Sollentuna, Sweden). In the experiments with the positive control, 1 × 10^4^ SH-SY5Y cells/wells were seeded in nontreated 24-well culture plates and treated with bezafibrate (Sigma), a pan-agonist of PPAR with EC_50_ of 50, 60, and 20 μM for PPARα, γ, and δ, respectively, for 7 days. The expansion was determined as described above.

### Data Analysis

The number *x* of independently repeated experiments is designated by “*n* = *x*”. Significant differences between mean values were determined by Wilcoxon’s signed rank-test of paired *t*-test, two-sided, where *p* values (*p < 0.05, **p < 0.01, ***p < 0.001, ****p < 0.0001) were considered statistically significant. Data are reported as means ± standard error of the mean (SEM). GraphPad Prism 9 (Graph Pad Software, San Diego, CA, USA) was used for analysis.

Concentrations of iso-α-acids and ethanol varied independently or in combination are presented in 3D surface response or 2D contour plots using SigmaPlot versions 12–14.

## Results

### Nonalcoholic and Alcoholic Beer Similarly Stimulate Growth of SH-SY5Y and NES Cells

**Figure 2:**
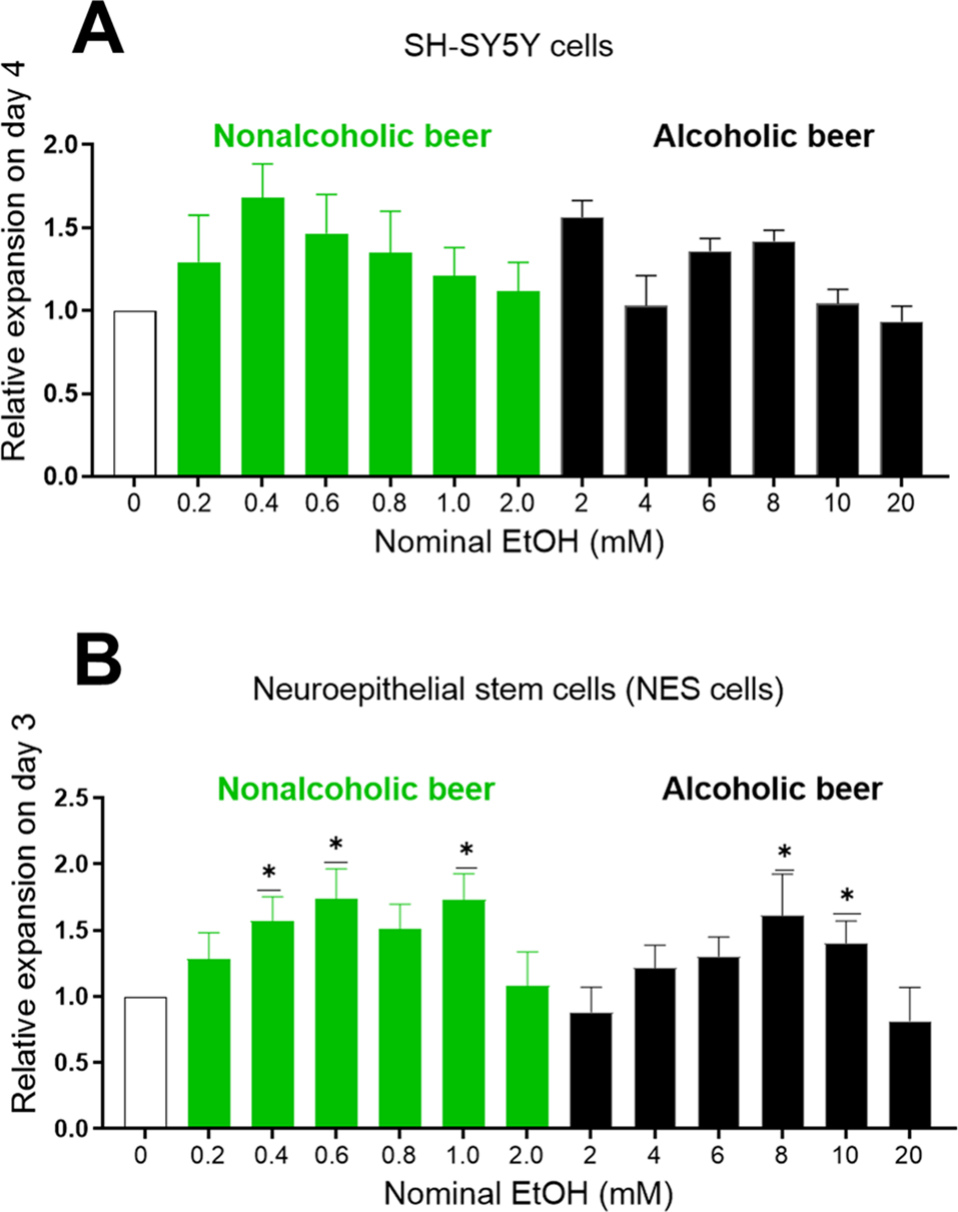
Similar expansion of SH-SY5Y and NES cells with nonalcoholic as with alcoholic beer. Relative expansion of (A) SH-SY5Y cells (*n* = 3) and (B) NES cells (*n* = 6) treated with nonalcoholic beer (Carlsberg Alcohol Free, 0.5% ABV), equivalent to 0.2–2 mM EtOH (green bars), or alcoholic beer (Carlsberg Export, 5.0% ABV), equivalent to 2–20 mM EtOH (black bars). Bars represent mean ± SEM. Friedman’s test shows that stimulation compared to controls was significantly different for both SH-SY5Y (*p* = 0.0253) and for NES cells (*p* = 0.0015). Using Wilcoxon’s two groups test, we find no statistically significant differences between any two groups of equal iso-α-acids content for either SH-SY5Y cells or NES cells without alcohol (nonalcoholic beer) compared with 10 times more alcohol (alcoholic beer). Values normalized to control experiments where no iso-α-acids were added (white bars). Statistics performed by Wilcoxon’s signed-rank test, two-sided, comparing each group with controls, *p < 0.05.

Using equivalent volumes of Carlsberg Alcohol Free (0.5% ABV, resulting in 0.2–2 mM ethanol) and Carlsberg Export (5% ABV, resulting in 2–20 mM ethanol), we observed that nonalcoholic and alcoholic beer similarly stimulate growth (expansion) of SH-SY5Y and NES cells (Figure 2A,B). The nominal concentrations of ethanol are shown in Figure 2.

Compared to the untreated controls both nonalcoholic and alcoholic beer significantly increase cell expansion. There are, however, no differences between groups with equal iso-α-acid but different alcohol concentrations. This result indicates that nonalcoholic beer contains at least one substance that stimulates the expansion of SH-SY5Y and NES cells. To test whether iso-α-acids could cause this effect, we examined the effect of iso-α-acids on expansion of neuron-like cell lines and NES cells in culture. We also examined if such effects could be enhanced in combination with ethanol.

### Iso-α-acids Stimulate Expansion of SH-SY5Y and NES Cells

**Figure 3:**
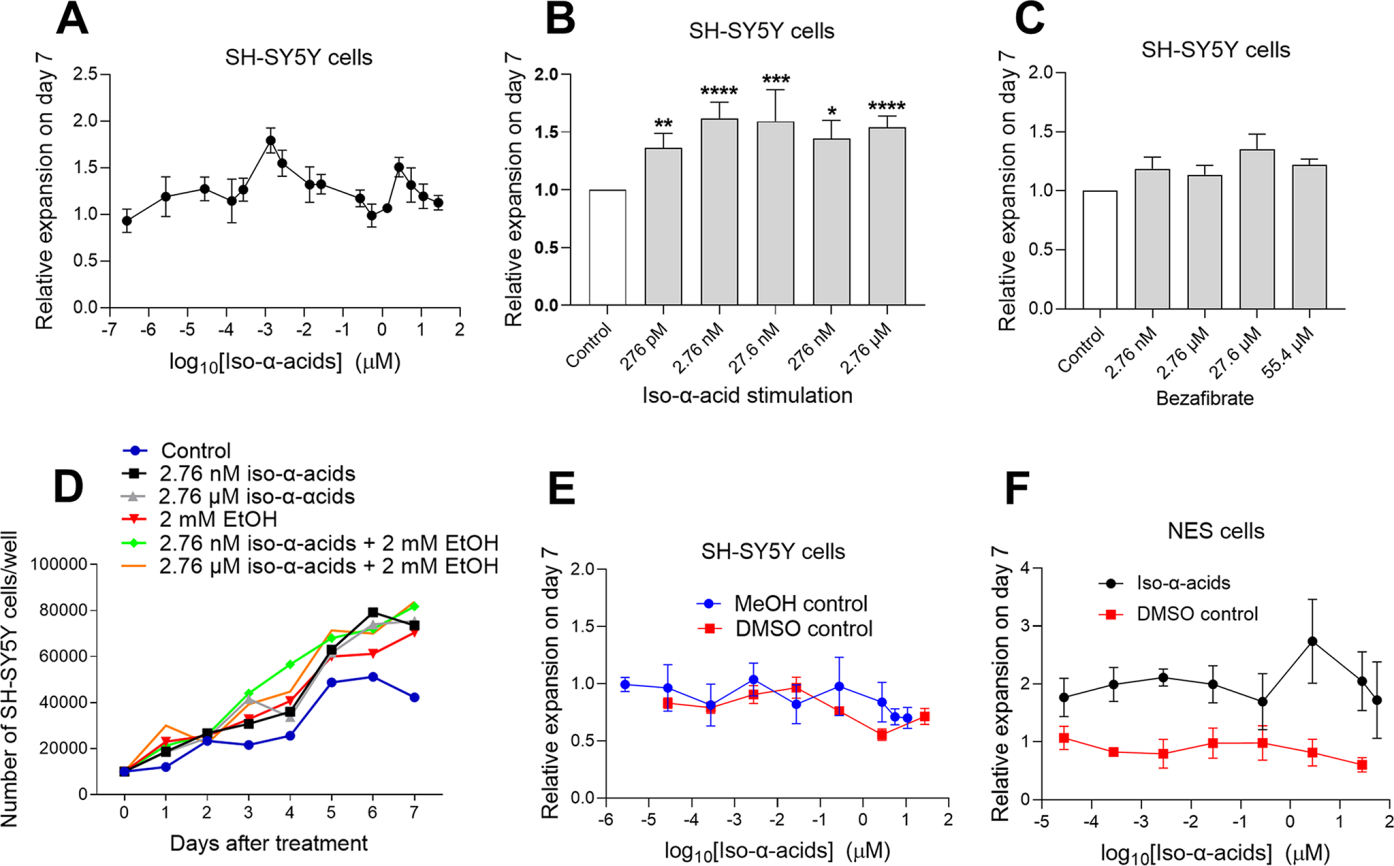
Expansion of SH-SY5Y and NES cells after treatment with iso-α-acids. (A) Relative expansion of SH-SY5Y cells after 7 days of treatment with different concentrations of iso-α-acids. Data represent mean ± SEM from up to 28 independent experiments. (B) Relative expansion of SH-SY5Y cells after 7 days of treatment with different concentrations of iso-α-acids: 276 pM (*n* = 22), 2.76 nM (*n* = 31), 27.6 nM (*n* = 29), 276 nM (*n* = 20), and 2.76 μM (*n* = 29). Values normalized to the control experiment where no iso-α-acids were added, “medium only” experiment (*n* = 38). Data represent mean ± SEM. Statistics performed by Wilcoxon’s signed-rank test, two-sided, comparing each group with controls, *p < 0.05, **p < 0.01, ***p < 0.001, ****p < 0.0001. (C) Relative expansion of SH-SY5Y cells after 7 days of treatment with different concentrations of bezafibrate (*n* = 3); data represent mean ± SEM. (D) Time course experiment over 7 days of SH-SY5Y cells treated day 0 without or with iso-α-acids without or with ethanol. Graph is representative of three independent experiments. (E) Effects of solvents on SH-SY5Y expansion. Data represent mean ± SEM from three independent experiments (*n* = 3) with either methanol, MeOH, or DMSO as controls. (F) Relative expansion of NES cells after 7 days of treatment with different concentrations of iso-α-acids or DMSO (control), *n* = 3–6; data represent mean ± SEM.

SH-SY5Y cells treated with different concentrations of iso-α-acids showed a bimodal dose–response relation with peaks at approximately 2–30 nM and 2–5 μM (Figure 3A). We performed repeated experiments and found that all treatments with iso-α-acids between 2.76 and 276 nM gave significantly higher expansion, *p* < 0.05 (Figure 3B). The highest mean ratio was found for 2.76 nM (mean ratio 1.6 ± 0.14, *p* < 0.0001, *n* = 31) and for 27.6 nM (mean ratio 1.6 ± 0.3, *p* = 0.0006, *n* = 29). Iso-α-acid stimulation was statistically significant and higher compared to the effect of the known PPAR agonist bezafibrate (Figure 3C). The kinetics of cell expansion using different treatments with iso-α-acids and ethanol showed increased cell expansion for 7 days (Figure 3D). The solvents MeOH or DMSO showed no effect per se (Figure 3E).

NES cells cultured for 7 days with iso-α-acids at different concentrations showed a bimodal dose–response relation with peaks at low nM and μM concentrations, and the solvent DMSO showed no effect (Figure 3F) .

**Figure 4:**
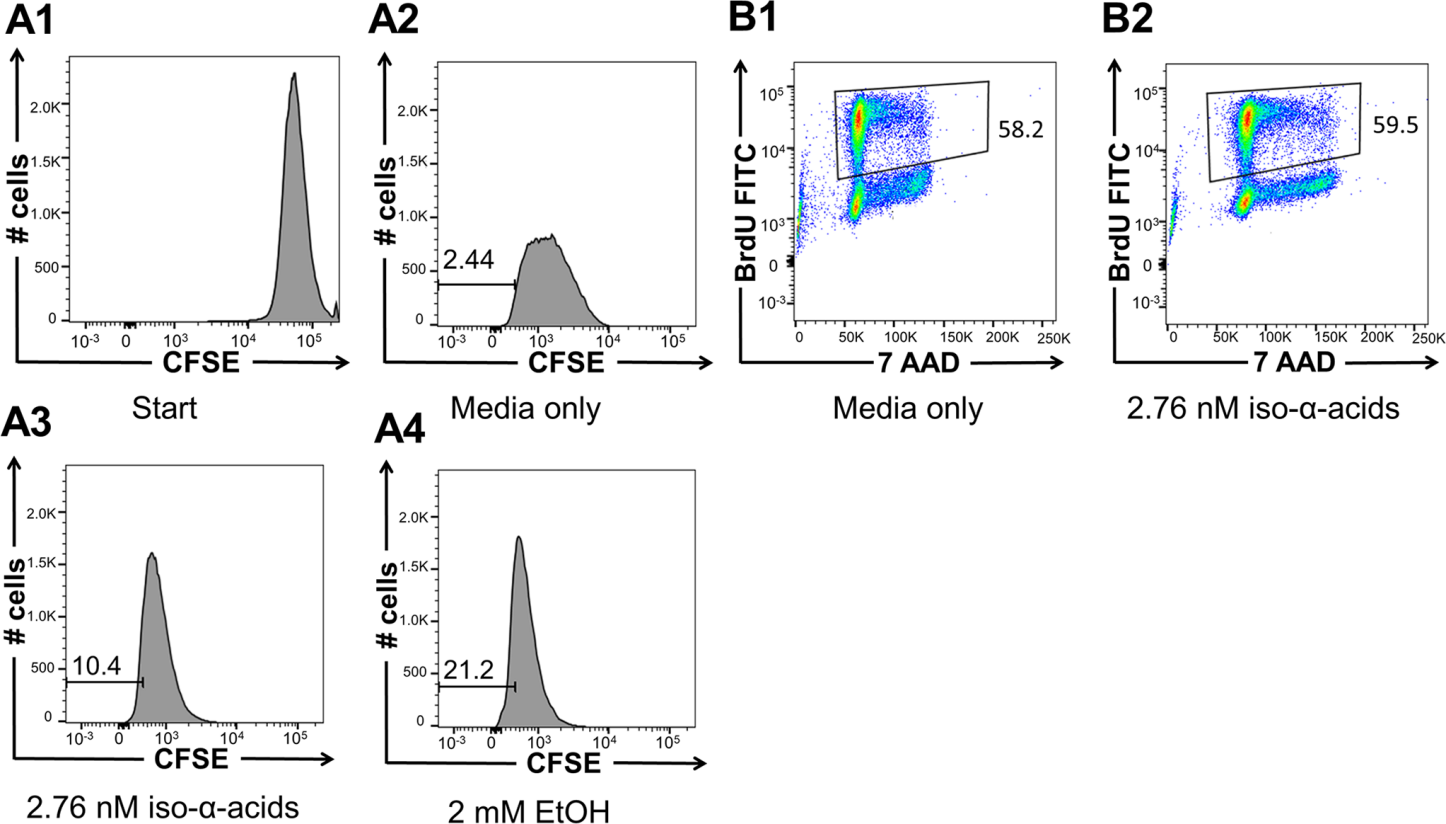
SH-SY5Y cell proliferation stimulated with iso-α-acids and ethanol. (A1–A4) Stimulated proliferation demonstrated using CFSE assays with media, iso-α-acids or ethanol, with proliferating SH-SY5Y cells indicated as CFSE^low^. (B1,B2) BrdU assay; stimulated proliferation of SH-SY5Y cells and relative proportion of proliferating cells in the S-phase with media or iso-α-acids indicated as 7AAD^+^ BrdU^+^ cells. The different graphs are representative of three or four independent experiments.

To quantify proliferation, SH-SY5Y cells were stained with carboxyfluorescein succinimidyl ester (CFSE). The number of cell divisions after 7 days was increased at 2.76 nM iso-α-acids compared with that of the control (Figure 4A). The increased number of SH-SY5Y cells in the S-phase after stimulation with iso-α-acids was also demonstrated with 5-bromo-2′deoxyuridine (BrdU) at 2.76 nM iso-α-acids (Figure 4B). These results indicate that iso-α-acids stimulate the expansion of SH-SY5Y and NES cells.

### Ethanol Stimulates Expansion of SH-SY5Y and NES Cells

**Figure 5:**
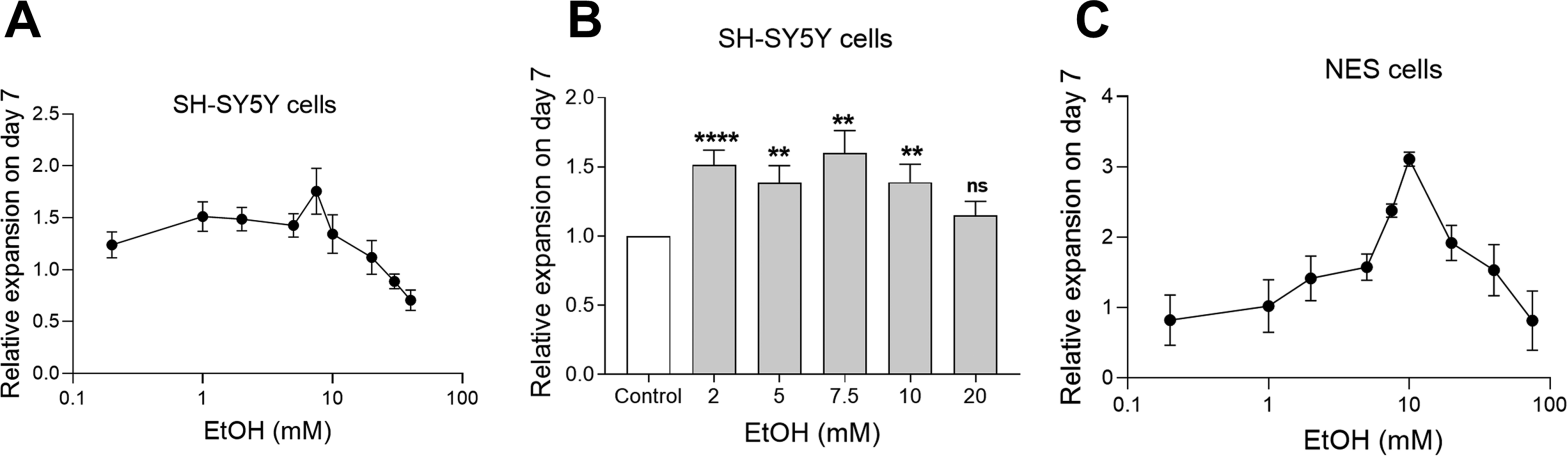
Expansion of SH-SY5Y and NES cells by ethanol. (A) Relative expansion of SH-SY5Y cells with ethanol in the concentration range of 0.2 to 40 mM, *n* = 7, data represent mean ± SEM. (B) Relative expansion of SH-SY5Y cells stimulated with different ethanol concentrations: 2 mM (*n* = 30), 5 mM (*n* = 17), 7.5 mM (*n* = 11), 10 mM (*n* = 19), 20 mM (*n* = 19). Values normalized to the control experiment where no ethanol was added, “medium only” (*n* = 34); data represent mean ± SEM. Statistics performed by Wilcoxon’s signed-rank test, two-sided, comparing each group with controls, *p < 0.05, **p < 0.01, ***p < 0.001, ****p < 0.0001; ns, equal no significant difference. (C) Relative expansion of NES cells stimulated with ethanol, *n* = 3; data represent mean ± SEM.

SH-SY5Y cells were incubated for 7 days with different concentrations of ethanol. We found that ethanol alone also stimulates cell expansion. The dose–response curve is bell-shaped, with a peak for ethanol concentration below 10 mM (Figure 5A).

Treatment with ethanol at concentrations of 2–10 mM gave a significantly higher expansion of SH-SY5Y cells (*p* < 0.05) (Figure 5B), and treatment with 7.5 mM gave the highest mean ratio 1.6 ± 0.2 (*p* = 0.0049, *n* = 11). To demonstrate proliferation, SH-SY5Y cells were stained with CFSE and treated with 2 mM ethanol for 7 days. The number of cell divisions measured as CFSE^low^ was higher for cells treated with ethanol compared to that with the control (Figure 4A).

NES cells treated for 7 days with ethanol showed that the dose–response curve is bell-shaped with a peak at around 10 mM ethanol (Figure 5C). These results indicate that ethanol on its own can stimulate expansion of both SH-SY5Y and NES cells.

### Ethanol Enhances Expansion of Iso-α-acid-Treated SH-SY5Y and N2a Cells at Certain Concentrations

As both iso-α-acids and ethanol stimulate the expansion of SH-SY5Y and NES cells, we asked what the combined effects would be. Accordingly, we treated SH-SY5Y and N2a cells with combinations of iso-α-acids and ethanol.

**Figure 6:**
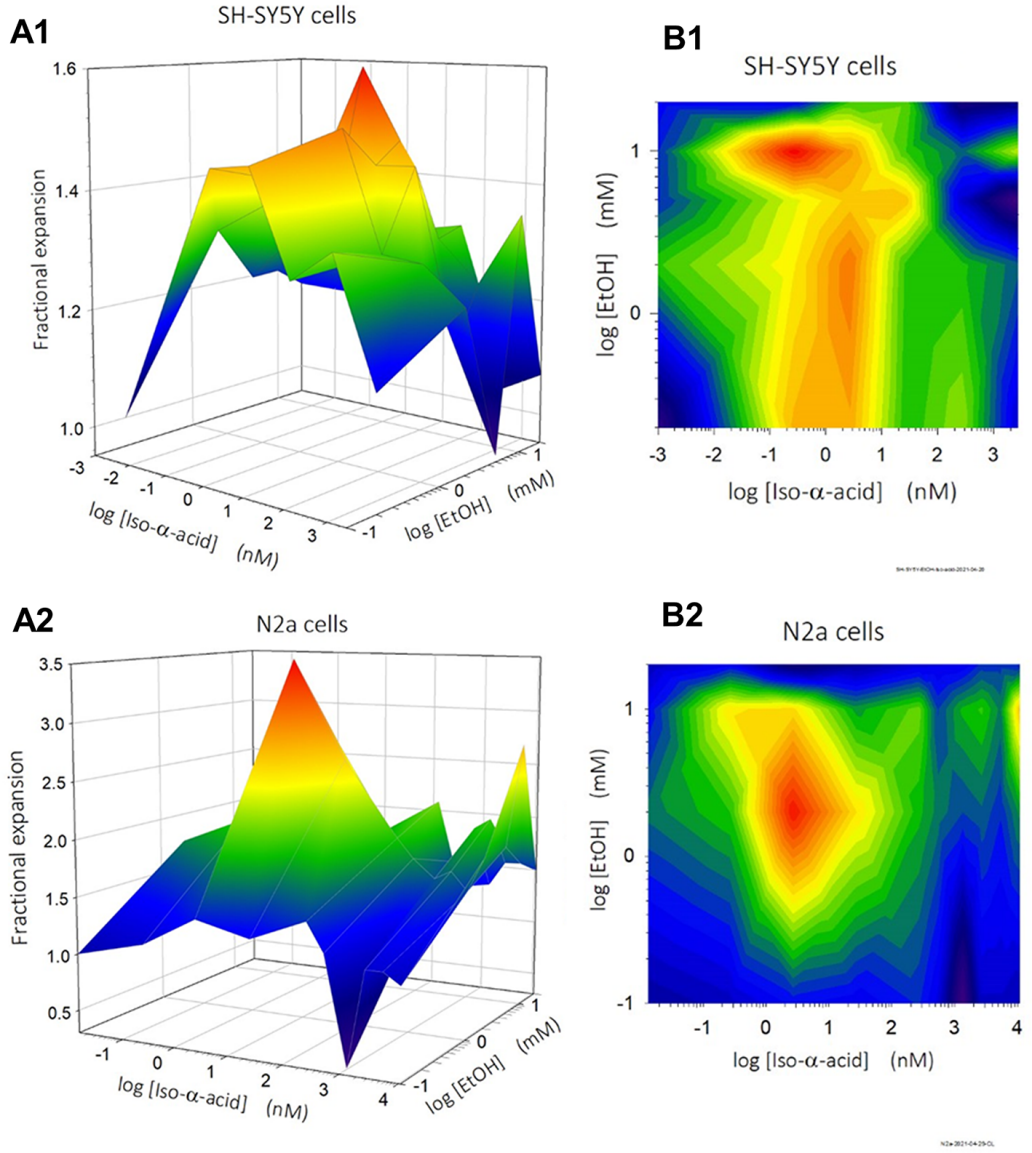
Enhanced expansion of SH-SY5Y and N2a cells after treatment with combinations of iso-α-acids and ethanol. Synergistic effects of iso-α-acids and ethanol on the expansion of SH-SY5Y (*n* = 7–10) and N2a cells (*n* = 3) presented in (A) 3D response surface plots and (B) 2D contour plots. Values are normalized to “medium only” (control) = 1. Most efficient expansion with combinations of iso-α-acids and ethanol concentrations is colored in red.

Figure 6 shows how independently varying the concentration of iso-α-acids and ethanol stimulates expansion of the SH-SY5Y and N2a cells, presented using both 3D surface plots and 2D contour plots.

The volumes of nonalcoholic beer (0.5% ABV) result in alcohol concentrations equivalent to 0.2–2 mM, and equivalent volumes of alcoholic beer (5% ABV) result in alcohol concentrations equivalent to 2–20 mM (Figure 2). The two beer types have the same bitterness of 18 IBU equivalent to 50 μM, and therefore, the different concentrations of iso-α-acids added with beer give the same concentrations of iso-α-acids, calculated to be between 117 nM to 1.17 μM.

As shown in Figure 6, the highest iso-α-acid-induced effect on SH-SY5Y and N2a cells is in the concentration range of 0.1 to 10 nM. Stimulation with iso-α-acids at 117 nM to 1.17 μM is more or less independent of the effects of alcohol at 0.1 to 10 mM EtOH (Figure 6)—concentrations relevant in beer drinking. This may also explain the observed lack of differences between alcoholic beer and nonalcoholic beer following addition to SH-SY5Y and NES cells.

### Iso-α-acids in Beer as an Explanation for the Expansion of SH-SY5Y and NES Cells with Beer

To further demonstrate the effects of iso-α-acids on cell expansion, SH-SY5Y and NES cells were treated with Carlsberg Alcohol Free (0.5% ABV) and Carlsberg Export (5% ABV) with volumes equivalent to iso-α-acid concentrations of 1 nM to 2.76 μM, yielding nominal EtOH concentrations of 0.002–4.8 mM for the nonalcoholic beer and 0.02–48 mM for the alcoholic beer.

**Figure 7:**
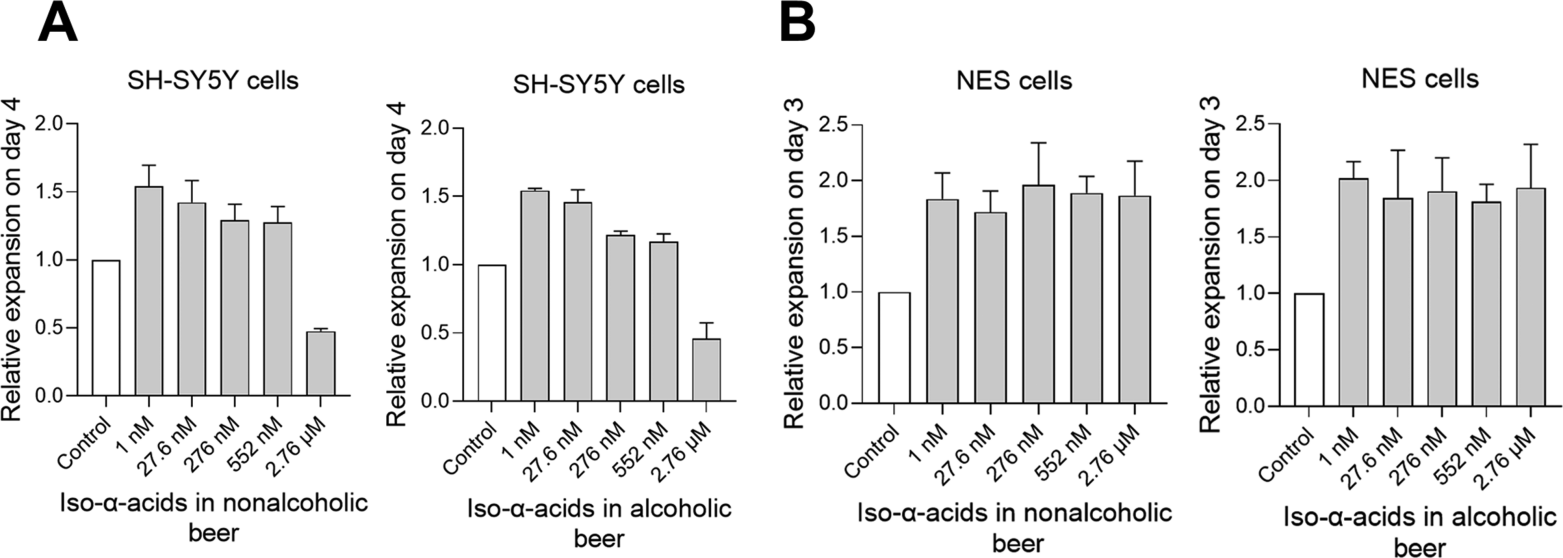
Iso-α-acids in nonalcoholic and alcoholic beer similarly stimulate expansion of SH-SY5Y and NES cells. Relative expansion of (A) SH-SY5Y cells (*n* = 3) and (B) NES cells (*n* = 3) treated with 1 nM to 2.76 μM of iso-α-acids from nonalcoholic beer (Carlsberg Alcohol Free, 0.5% ABV) equivalent to 0.002–4.76 mM EtOH and alcoholic beer (Carlsberg Export, 5.0% ABV), equivalent to 0.02–47.6 mM EtOH. Friedman’s test was used. Groups with increasing alcohol and iso-α-acid concentrations showed significant stimulation compared to controls for both SH-SY5Y (*p* = 0.0081) and NES cells (*p* = 0.0081). Using Wilcoxon’s test, we found no difference, however, between groups for either SH-SY5Y or NES cells when comparing the same amounts of iso-α-acids without alcohol (nonalcoholic beer) with 10 times more alcohol (alcoholic beer).

As shown in Figure 7, SH-SY5Y cells and NES cells expand when treated with either of the two beer types and with different concentrations of iso-α-acids, confirming our results in Figure 3A,F.

### Effects of *Trans*-iso-α-acids on Expansion of SH-SY5Y and NES Cells

In beer, there are different iso-forms of iso-α-acids as well as reduced forms of iso-α-acids. To investigate the effects of α-acids and their derivatives on cell growth, different cell lines were treated for 7 days with different forms of iso-α-acids, as listed in Table S1, Supporting Information.

**Figure 8:**
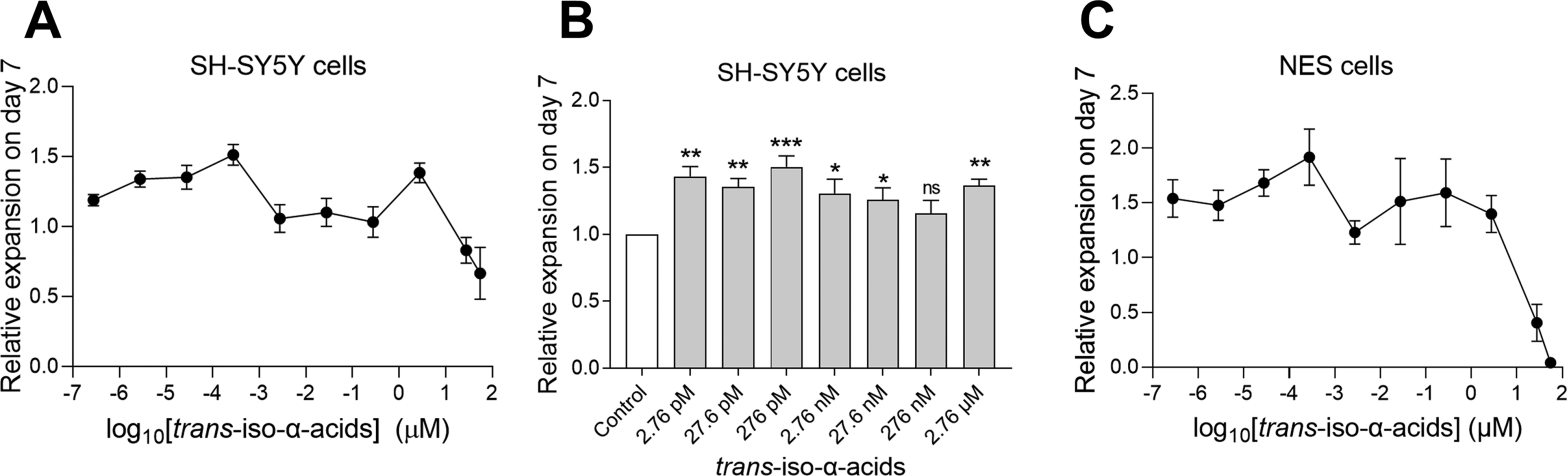
Expansion of SH-SY5Y and NES cells with *trans*-iso-α-acids. (A) Relative expansion of SH-SY5Y cells, treated with *trans*-iso-α-acids for 7 days, *n* = 3–5; data represent mean ± SEM. (B) Relative expansion of SH-SY5Y cells treated with *trans*-iso-α-acids at different concentrations: 2.76 pM (*n* = 7), 27.6 pM (*n* = 7), 276 pM (*n* = 8), 2.76 nM (*n* = 10), 27.6 nM (*n* = 10), 276 nM (*n* = 5), and 2.76 μM (*n* = 5). Values normalized to “medium only” control (*n* = 10); data represent mean ± SEM. Statistics performed by paired *t*-test, two-sided, comparing each group with controls, *p < 0.05, **p < 0.01, ***p < 0.001; ns, equal no significant difference. (C) Relative expansion of NES cells, *n* = 4; data represent mean ± SEM.

Treatment with *trans*-iso-α-acids showed a bimodal bell-shaped dose–response curve like those with iso-α-acids, although incubating with *trans*-iso-α-acids gave a peak at a lower concentration (Figure 8A). Treatment with *trans*-iso-α-acids resulted in significantly greater expansion of SH-SY5Y cells at 2.76 pM to 27.6 nM and 2.76 μM (*p* < 0.05) (Figure 8B), and treatment with 276 pM gave the highest mean ratio of 1.5 ± 0.08 (*p* = 0.0005, *n* = 8). NES cells treated with *trans*-iso-α-acids also yielded a bimodal dose–response curve with a peak at a lower concentration, (Figure 8C). These results suggest that the *trans*-form has a higher potency compared with the *cis*-form.

### PPARα, PPARγ, and CB_1_ Antagonist Effects on *Trans*-iso-α-acid-Stimulated SH-SY5Y Cells

**Figure 9:**
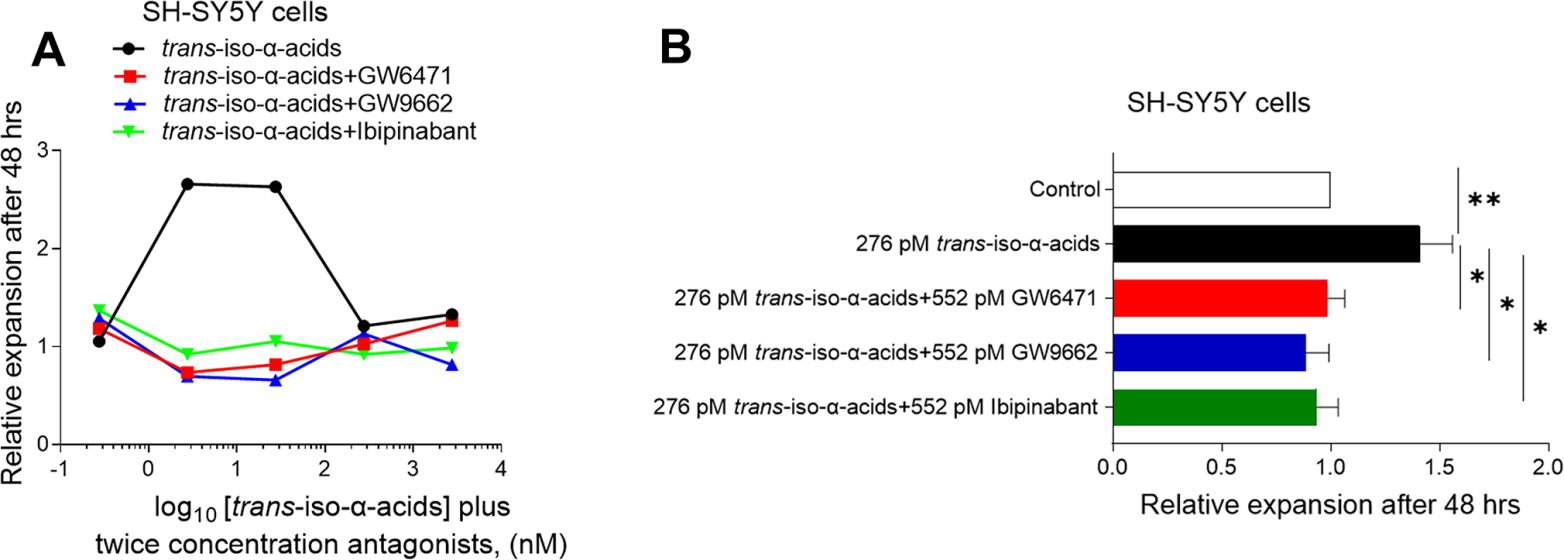
Effects of antagonists on expansion of *trans*-iso-α-acid stimulated SH-SY5Y cells. (A) Relative expansion of SH-SY5Y cells treated with *trans*-iso-α-acids and twice the concentration of antagonists for PPARα (GW6471), PPARγ (GW9662), and CB_1_ (Ibipinabant) for 48 h; data are representative of three different experiments. (B) Relative expansion of SH-SY5Y cells stimulated with 276 pM *trans*-iso-α-acids (*n* = 8) together with 552 nM of antagonists of PPARα (*n* = 7), PPARγ (*n* = 7), or CB_1_ (*n* = 8) for 48 h. Values normalized to “medium only” controls (*n* = 8); data represent mean ± SEM. Statistics performed by Wilcoxon’s signed-rank test, two-sided, comparing each group with controls, *p < 0.05, **p < 0.01.

Iso-α-acids act via the peroxisome proliferator-activated receptors,^10^ and since *trans*-iso-α-acids most potently activate SH-SY5Y cells, we investigated if PPARα and PPARγ mediate the effects at the low concentration measured. As hops belong to the Cannabaceae family, we likewise investigated if the cannabinoid receptor CB_1_, upstream in PPAR signaling,^11^ is involved in the observed cell expansion. SH-SY5Y cells were stimulated for 48 h with 276 pM to 2.76 μM *trans*-iso-α-acids together with twice the concentration of the PPARα antagonist GW6471, PPARγ antagonist GW9662, and the cannabinoid receptor CB_1_ antagonist Ibipinabant. Treatment with 2.76 and 27.6 nM *trans*-iso-α-acid led to increased cell expansion as compared to media alone (Figure 9A). Expansion was inhibited by either the PPARα antagonist GW6471, the PPARγ antagonist GW9662, or the cannabinoid receptor CB_1_ antagonist Ibipinabant (*p* < 0.05) (Figure 9B). These results indicate that PPARα, PPARγ, and CB_1_ mediate the effects of *trans*-iso-α-acids at low nM concentrations.

## Discussion

Our study indicates that nonalcoholic beer can stimulate expansion of neuroepithelial stem cells in culture, and that iso-α-acids from the hop plant could possibly explain this expansion. Alcoholic and nonalcoholic beer give effects similar to those of iso-α-acids alone, which supports our conclusions. We show further that iso-α-acids can stimulate SH-SY5Y and neuroepithelial stem cells and do so within a nanomolar concentration range relevant to concentrations reached in beer drinking. Concentrations of *trans*-iso-α-acids reach approximately 40 nM in blood 30 min after drinking two bottles of low-hopped beer^14^ and to around 150 nM in post-mortem cases from people who had been drinking beer.^15^ Iso-α-acids can also pass the blood–brain barrier.^6^

An alcohol range of less than 10 mM, which on its own can expand SH-SY5Y and neuroepithelial stem cells, is also relevant to what is found with moderate alcohol intake. Thirty minutes after drinking two bottles of alcoholic beer (5.3% ABV), the blood alcohol concentration is 32 mg/100 mL, and two bottles would equal around 6–7 mM.^14^ We have earlier shown that around 2.5 mM ethanol can enhance the expansion of γδ T cells stimulated with isopentenylpyrophosphate.^16^ We expected to find an enhancing effect of alcohol on iso-α-acid-stimulated SH-SY5Y and NES cells, as well. Surprisingly, there was no enhancing effect in the concentration range relevant for beer drinking, and this can explain why the effects on cell expansion are similar for nonalcoholic and alcoholic beer (Figure 6).

Once absorbed, alcohol is distributed in the body, including the brain, as ethanol passes the blood–brain barrier easily. The distribution is approximately 0.6 L/kg for women and 0.7 L/kg for men.^17^ The volume of beer equivalent to iso-α-acid concentration in Figure 2 was calculated. Thus, for a person with a weight of 80 kg, nonalcoholic beer around 400–700 mL, or around 1–2 bottles, would give the greatest effect on the expansion of NES cells.

Iso-α-acids in beer are a mixture of at least six different stereoisomers (Figure 1), and there are also other forms of reduced iso-α-acids. We tried to analyze whether one or more of the forms of iso-α-acids, either the *cis/trans* stereoisomers or any of the reduced forms, have effects on expansion. We found that, compared with a mixture of iso-α-acids, the *trans*-form activates SH-SY5Y and NES cells at a lower concentration. Compared to *cis*-iso-α-acids, *trans*-iso-α-acids are less soluble in water and are therefore present to a greater extent in the foam of beer.^18^ The more lipophilic *trans*-form may penetrate more easily through the cell membrane or bind with higher affinity to receptors involved. Both the *cis*- and *trans*-iso-α-acids are formed from isomerization of α-acids during wort boiling in the brewing process. The *cis/trans* ratio in wort is usually 68:32,^19^ and this ratio is likely unchanged with consumption.

Iso-α-acids have been shown to be ligands of the nuclear receptors PPARγ/PPARα and to bind at 3–30 μM concentrations.^10^ Here, we show that the PPARγ/PPARα and the cannabinoid receptor CB_1_ may be activated by iso-α-acids at the lower (nM) concentrations (Figure 9), an iso-α-acid concentration present in beer drinking. Neurite outgrowth at 100 nM to 1 μM concentrations of PPARγ agonists has been found in SH-SY5Y cells.^20^ Other studies have also found growth of neural stem cells at low μM concentration,^21^ observations that support the physiological relevance of our findings.

Iso-α-acids as PPARγ ligands can cause a metabolic reprogramming of microglia to suppress inflammation.^6^ Iso-α-acids may also be important for metabolic reprogramming during adult neurogenesis.^22^ Proliferation requires an upregulation of aerobic glycolysis, while differentiation needs a metabolic switch to the more efficient oxidative phosphorylation. Ligands of PPARγ have been shown to induce neural stem cell proliferation,^21,23^ whereas ligands of PPARα enhance differentiation of astrocytes,^24^ PPARγ increases oligodendrocytes,^25^ and PPARα expands neurons.^26^ As iso-α-acids in beer are both PPARα and PPARγ ligands, these compounds may not only generate proliferation of neuroepithelial stem cells but also induce differentiation.

The significance of consuming bitter compounds, as for instance hop products, has attracted renewed interest in recent years after characterization of the chemosensory tuft cells equipped with taste receptors TAS2Rs^27^ present throughout the digestive tract.^28^ It has recently been demonstrated that the bitter taste compounds, including iso-α-acids, are involved in hormone release from enteroendocrine cells,^29^ and they signal through tuft cells to the immune system.^30^ The stereoselectivity for iso-α-acids of TAS2Rs is, however, different from what we have observed here, where the *cis*-form rather than the *trans*-form has a higher potency at certain TAS2Rs.^31^

It is intriguing that beer has recently been shown to protect against amyloid β aggregation in the brain.^32^ In Alzheimer’s disease (AD), it is not only the number but also the maturation of neurons that decline.^33^ Nonalcoholic beer has been shown to stimulate the number of circulating endothelial progenitor cells.^34^ If nonalcoholic beer also can stimulate expansion of neuroepithelial stem cells, this can be one explanation of the neuroprotective effect of beer. Other components in beer from hops such as xanthohumol improve cognitive flexibility^35^ and stimulate neurite outgrowth.^36^ Hop components have been used in traditional medicine for over a thousand years. These compounds are safe and without side effects at physiologically relevant concentrations, and they may therefore be suitable as food additives.^37^

In conclusion, our results using neuroepithelial stem cells indicate that nonalcoholic beer with ingredients such as iso-α-acids have many of the beneficial effects of alcoholic beer.

## Supporting Information Available

HPLC of Hopsteiner Iso-Extract, RPLC-MS, and UV of purified iso-α-acids; structures of the reduced iso-α-acids tested; concentration ranges of iso-α-acids and ethanol in experiments (PDF)

## References

[ref1] de Gaetano G.; et al. Effects of moderate beer consumption on health and disease: A consensus document. Nutr., Metab. Cardiovasc. Dis. 2016, 26 (6), 443–67. 10.1016/j.numecd.2016.03.007. 27118108

[ref2] Hagemann M. H.; et al. Chances for dry-hopped non-alcoholic beverages? Part 1: Concept and market prospects. Brewing Science 2016, 69, 50–55.

[ref3] Karabin M.; et al. Biologically Active Compounds from Hops and Prospects for Their Use. Compr. Rev. Food Sci. Food Saf. 2016, 15 (3), 542–567. 10.1111/1541-4337.12201. 33401815

[ref4] Van Cleemput M.; et al. Hop (Humulus lupulus)-derived bitter acids as multipotent bioactive compounds. J. Nat. Prod. 2009, 72 (6), 1220–30. 10.1021/np800740m. 19476340

[ref5] Koetter U.; Biendl M. Hops (Humulus lupulus): A review of its Historic and Medicinal Uses. Herbal Gram 2010, 87, 44–57.

[ref6] Ano Y.; et al. Iso-alpha-acids, Bitter Components of Beer, Prevent Inflammation and Cognitive Decline Induced in a Mouse Model of Alzheimer’s Disease. J. Biol. Chem. 2017, 292 (9), 3720–3728. 10.1074/jbc.M116.763813. 28087694 PMC5339755

[ref7] Ano Y.; et al. Iso-alpha-acids, Hop-Derived Bitter Components of Beer, Attenuate Age-Related Inflammation and Cognitive Decline. Front. Aging Neurosci. 2019, 11, 16 10.3389/fnagi.2019.00016. 30778295 PMC6369178

[ref8] Ano Y.; et al. Iso-alpha-acids, the bitter components of beer, improve hippocampus-dependent memory through vagus nerve activation. FASEB J. 2019, 33 (4), 4987–4995. 10.1096/fj.201801868RR. 30601670 PMC6436653

[ref9] Ano Y.; et al. Iso-alpha-Acids, Bitter Components in Beer, Suppress Inflammatory Responses and Attenuate Neural Hyperactivation in the Hippocampus. Front. Pharmacol. 2019, 10, 81 10.3389/fphar.2019.00081. 30804789 PMC6378368

[ref10] Yajima H.; et al. Isohumulones, bitter acids derived from hops, activate both peroxisome proliferator-activated receptor alpha and gamma and reduce insulin resistance. J. Biol. Chem. 2004, 279 (32), 33456–62. 10.1074/jbc.M403456200. 15178687

[ref11] O’Sullivan S. E. Cannabinoids go nuclear: evidence for activation of peroxisome proliferator-activated receptors. Br. J. Pharmacol. 2007, 152 (5), 576–82. 10.1038/sj.bjp.0707423. 17704824 PMC2190029

[ref12] Uhlin E.; et al. Derivation of human iPS cell lines from monozygotic twins in defined and xeno free conditions. Stem Cell Res. 2017, 18, 22–25. 10.1016/j.scr.2016.12.006. 28395796

[ref13] Lundin A.; et al. Human iPS-Derived Astroglia from a Stable Neural Precursor State Show Improved Functionality Compared with Conventional Astrocytic Models. Stem Cell Rep. 2018, 10 (3), 1030–1045. 10.1016/j.stemcr.2018.01.021. PMC5918339 29456185

[ref14] Rodda L. N.; Gerostamoulos D.; Drummer O. H. , *Pharmacokinetics of iso-alpha-acids in volunteers following the consumption of beer.*. J. Anal. Toxicol. 2014, 38 (6), 354–9. 10.1093/jat/bku038. 24778090

[ref15] Rodda L. N.; Gerostamoulos D.; Drummer O. H. Detection of iso-alpha-acids to confirm beer consumption in postmortem specimens. Drug Test. Anal. 2015, 7 (1), 65–74. 10.1002/dta.1749. 25421420

[ref16] Laurent A. J.; et al. Synergistic effects of ethanol and isopentenyl pyrophosphate on expansion of gammadelta T cells in synovial fluid from patients with arthritis. PLoS One 2014, 9 (8), e103683 10.1371/journal.pone.0103683. 25090614 PMC4121167

[ref17] Maskell P. D.; et al. Evidence based survey of the distribution volume of ethanol: Comparison of empirically determined values with anthropometric measures. Forensic Sci. Int. 2019, 294, 124–131. 10.1016/j.forsciint.2018.10.033. 30553124

[ref18] Hughes P. The significance of Iso-α-Acids for Beer Quality Cambridge Prize Paper. J. Inst. Brew. 2000, 106 (5), 271–276. 10.1002/j.2050-0416.2000.tb00066.x.

[ref19] De Keukeleire D. Fundamentals of beer and hop chemistry. Quim. Nova 2000, 23 (1), 108–112. 10.1590/S0100-40422000000100019.

[ref20] Miglio G.; et al. PPARgamma stimulation promotes mitochondrial biogenesis and prevents glucose deprivation-induced neuronal cell loss. Neurochem. Int. 2009, 55 (7), 496–504. 10.1016/j.neuint.2009.05.001. 19442697

[ref21] Wada K.; et al. Peroxisome proliferator-activated receptor gamma-mediated regulation of neural stem cell proliferation and differentiation. J. Biol. Chem. 2006, 281 (18), 12673–81. 10.1074/jbc.M513786200. 16524877

[ref22] Di Giacomo E.; et al. Roles of PPAR transcription factors in the energetic metabolic switch occurring during adult neurogenesis. Cell Cycle 2017, 16 (1), 59–72. 10.1080/15384101.2016.1252881. 27860527 PMC5270516

[ref23] Morales-Garcia J. A.; et al. Peroxisome proliferator-activated receptor gamma ligands regulate neural stem cell proliferation and differentiation in vitro and in vivo. Glia 2011, 59 (2), 293–307. 10.1002/glia.21101. 21125653

[ref24] Cristiano L.; et al. Peroxisome proliferator-activated receptors (PPARs) and related transcription factors in differentiating astrocyte cultures. Neuroscience 2005, 131 (3), 577–87. 10.1016/j.neuroscience.2004.11.008. 15730864

[ref25] Roth A. D.; et al. PPAR gamma activators induce growth arrest and process extension in B12 oligodendrocyte-like cells and terminal differentiation of cultured oligodendrocytes. J. Neurosci. Res. 2003, 72 (4), 425–35. 10.1002/jnr.10596. 12704804

[ref26] Bento-Abreu A.; Tabernero A.; Medina J. M. Peroxisome proliferator-activated receptor-alpha is required for the neurotrophic effect of oleic acid in neurons. J. Neurochem. 2007, 103 (3), 871–81. 10.1111/j.1471-4159.2007.04807.x. 17683485

[ref27] Luo M.; Ni K.; Jin Y.; Yu Z.; Deng L. Toward the Identification of Extra-Oral TAS2R Agonists as Drug Agents for Muscle Relaxation Therapies via Bioinformatics-Aided Screening of Bitter Compounds in Traditional Chinese Medicine. Front. Physiol. 2019, 10, 861 10.3389/fphys.2019.00861. 31379593 PMC6647893

[ref28] Prandi S.; et al. Expression profiling of Tas2r genes reveals a complex pattern along the mouse GI tract and the presence of Tas2r131 in a subset of intestinal Paneth cells. Cell. Mol. Life Sci. 2018, 75 (1), 49–65. 10.1007/s00018-017-2621-y. 28801754 PMC11105753

[ref29] Yamazaki T.; et al. Bitter taste receptor activation by hop-derived bitter components induces gastrointestinal hormone production in enteroendocrine cells. Biochem. Biophys. Res. Commun. 2020, 533 (4), 704–709. 10.1016/j.bbrc.2020.10.099. 33160623

[ref30] Xiong X.; et al. Berberine in the treatment of ulcerative colitis: A possible pathway through Tuft cells. Biomed. Pharmacother. 2021, 134, 111129 10.1016/j.biopha.2020.111129. 33348308

[ref31] Intelmann D.; et al. Three TAS2R Bitter Taste Receptors Mediate the Psychophysical Responses to Bitter Compounds of Hops (Humulus lupulus L.) and Beer. Chemosens. Percept. 2009, 2 (3), 118–132. 10.1007/s12078-009-9049-1.

[ref32] Kok E. H.; et al. Beer Drinking Associates with Lower Burden of Amyloid Beta Aggregation in the Brain: Helsinki Sudden Death Series. Alcohol.: Clin. Exp. Res. 2016, 40 (7), 1473–8. 10.1111/acer.13102. 27218874

[ref33] Moreno-Jimenez E. P.; et al. Adult hippocampal neurogenesis is abundant in neurologically healthy subjects and drops sharply in patients with Alzheimer’s disease. Nat. Med. 2019, 25 (4), 554–560. 10.1038/s41591-019-0375-9. 30911133

[ref34] Chiva-Blanch G.; et al. The non-alcoholic fraction of beer increases stromal cell derived factor 1 and the number of circulating endothelial progenitor cells in high cardiovascular risk subjects: a randomized clinical trial. Atherosclerosis 2014, 233 (2), 518–24. 10.1016/j.atherosclerosis.2013.12.048. 24530788

[ref35] Zamzow D. R.; et al. Xanthohumol improved cognitive flexibility in young mice. Behav. Brain Res. 2014, 275, 1–10. 10.1016/j.bbr.2014.08.045. 25192637 PMC4281268

[ref36] Oberbauer E.; et al. Chroman-like cyclic prenylflavonoids promote neuronal differentiation and neurite outgrowth and are neuroprotective. J. Nutr. Biochem. 2013, 24 (11), 1953–62. 10.1016/j.jnutbio.2013.06.005. 24070601

[ref37] Sasaoka N.; et al. Long-term oral administration of hop flower extracts mitigates Alzheimer phenotypes in mice. PLoS One 2014, 9 (1), e87185 10.1371/journal.pone.0087185. 24489866 PMC3906130

