# Supplementary material for: Iso-α-acids in Nonalcoholic and Alcoholic Beer Stimulate Growth of Neuron-like SH-SY5Y Cells and Neuroepithelial Stem Cells
Source: ACS Bio Med Chem Au. 2021 Sep 7;1(1):11–20. doi: 10.1021/acsbiomedchemau.1c00017 (PMC10125168; doi:10.1021/acsbiomedchemau.1c00017)
Supplement: Supplementary file 1 — bg1c00017_si_001.pdf [file bg1c00017_si_001.pdf]

Supporting Information

Iso-α-acids in Nonalcoholic and Alcoholic Beer Stimulate Growth of Neuron-like SH-SY5Y Cells and Neuroepithelial Stem Cells

Agneta J. Laurent[a]*, Niels Bindslev[b], Vladana Vukojević[a], Lars Terenius[a]

[a]*Department of Clinical Neuroscience, Center for Molecular Medicine, Karolinska Institutet, SE-171 76 Stockholm, Sweden*

[b]*Department of Biomedical Sciences, Faculty of Health and Medical Sciences, University of Copenhagen, Blegdamsvej 3B, DK-2200 Copenhagen N, Denmark*

---

*Corresponding author:

Agneta Laurent, Department of Clinical Neuroscience, Center for Molecular Medicine, Karolinska Institutet, SE-171 76 Stockholm, Sweden. Email: agneta.laurent@ki.se

Mobile phone: +46 76 2064135

Table of Contents

Fig. S1	HPLC chromatogram for Hopsteiner Iso-Extract	2
Fig. S2	HPLC analysis report Hopsteiner Iso-Extract	3
Fig. S3	RPLC-MS and UV of purified iso-α-acids	4
Fig. S4	Structures of the reduced iso-α-acids tested	5
Table S1	Concentration ranges of iso-α-acids and ethanol in experiments	6

```
mple Name: Hopsteiner HPLC

=====================================================================
Acq. Operator   :                                  Seq. Line :   1
Acq. Instrument : Instrument 1                      Location :   21
Injection Date  : 2018-06-28 16:55:19                    Inj :   1
                                                 Inj Volume : Inj prog
Acq. Method     : C:\HPCHEM\1\METHODS\STANDARD\B1090A3.M
Last changed    : 2013-10-17 22:01:52 by Doris_rg
Analysis Method : C:\USERS\PUBLIC\DOCUMENTS\CHEMSTATION\1\METHODS\Newtest.M
Last changed    : 2021-03-16 11:41:57 by SYSTEM
                  (modified after loading)
Method Info     : Gradient method for natural product extract
                  Water/acetonitrile 95:5 -> 5:95 in 10 minutes (5:95 for 2 min)
                  Injection volume: 2 µL
                  Operator: Udo Huber
```

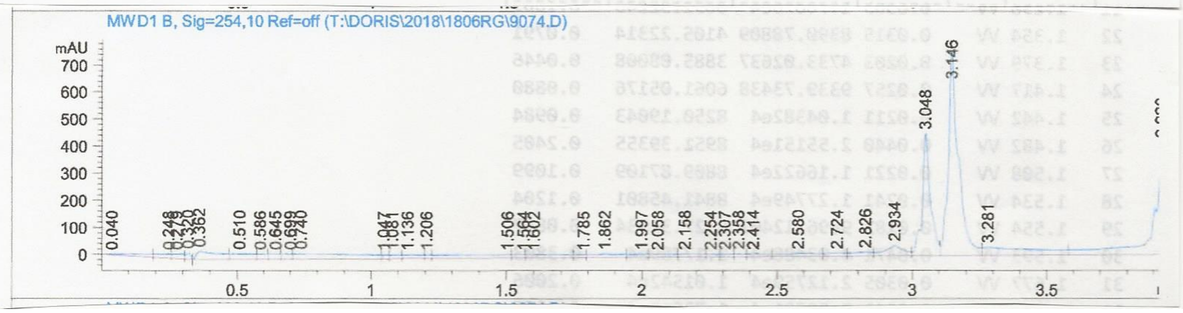

Figure S1. High performance liquid chromatography (HPLC) chromatogram for Hopsteiner Iso-Extract 30 %.

mple Name: Hopsteiner HPLC

Signal 3: MWD1 B, Sig=254,10 Ref=off

Peak #	RetTime [min]	Type	Width [min]	Area [mAU*s]	Height [mAU]	Area %
1	0.040	BV	0.2791	65.99284	2.78869	0.8991
2	0.248	VV	0.0471	54.23235	15.01619	0.7389
3	0.279	VV	0.0382	48.17196	16.90540	0.6563
4	0.320	VV	0.0186	25.19744	21.54585	0.3433
5	0.362	VV	0.0757	166.02063	28.20208	2.2619
6	0.510	VV	0.0720	117.14866	19.52803	1.5960
7	0.586	VV	0.0443	49.80950	18.71974	0.6786
8	0.645	VV	0.0345	52.05788	18.67109	0.7092
9	0.699	VV	0.0389	48.45561	18.26984	0.6602
10	0.740	VV	0.2025	313.82083	18.26344	4.2755
11	1.047	VV	0.0216	22.35944	13.31142	0.3046
12	1.081	VV	0.0461	35.47641	12.83384	0.4833
13	1.136	VV	0.0635	62.02942	12.59168	0.8451
14	1.206	VV	0.0257	18.91581	12.27363	0.2577
15	1.506	VV	0.2095	203.01045	11.58013	2.7658
16	1.564	VV	0.0419	34.54512	11.55131	0.4706
17	1.602	VV	0.1221	116.60407	11.52279	1.5886
18	1.785	VV	0.0387	29.22535	10.42185	0.3982
19	1.862	VV	0.0995	91.31889	11.39248	1.2441
20	1.997	VV	0.0586	49.52723	10.38899	0.6748
21	2.058	VV	0.0760	62.73390	10.46186	0.8547
22	2.158	VV	0.0701	61.95390	11.11071	0.8441
23	2.254	VV	0.0382	26.35210	8.99002	0.3590
24	2.307	VV	0.0376	27.38078	9.22446	0.3730
25	2.358	VV	0.0352	25.51596	9.53436	0.3476
26	2.414	VV	0.0638	49.65498	10.02161	0.6765
27	2.580	VV	0.1141	100.09000	10.51163	1.3636
28	2.724	VV	0.0662	52.14094	10.83202	0.7104
29	2.826	VV	0.0605	66.21645	14.17737	0.9021
30	2.934	VV	0.0625	176.83870	37.86275	2.4093
31	3.048	VV	0.0304	979.11975	450.30316	13.3396
32	3.146	VV	0.0351	1966.10071	760.98059	26.7863
33	3.281	VV	0.1724	431.91321	30.10616	5.8844
34	3.928	VBA	0.0518	1710.00415	417.77744	23.2973
Totals :				7339.93540	2087.67257	

Figure S2. HPLC analysis report for Hopsteiner Iso-Extract 30 % (UV detection at 254 nm)

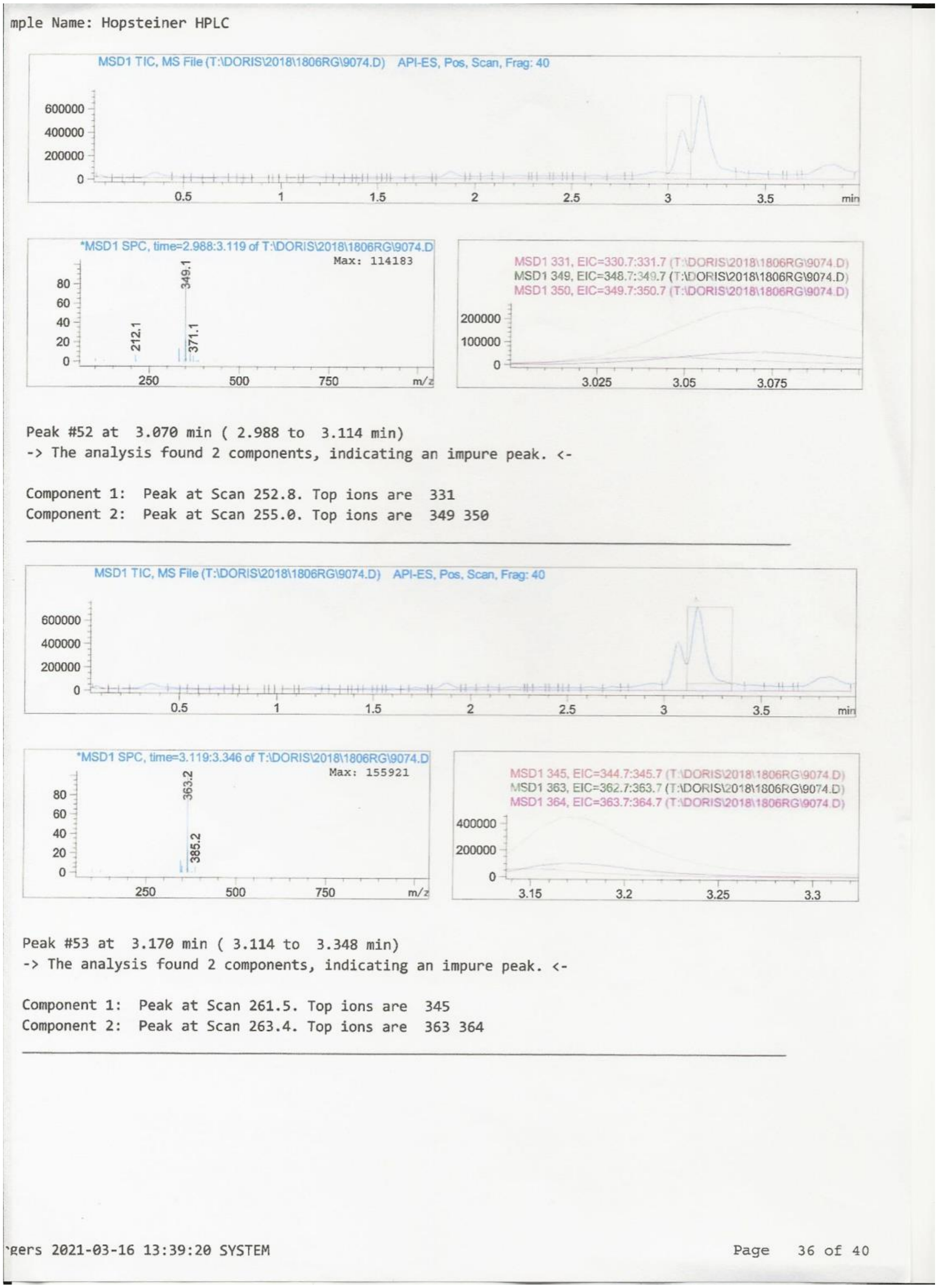

Figure S3. Reversed-phase liquid chromatography mass-spectrometry **(**RPLC-MS) and UV detection of purified iso-α-acids collected under the peaks with maximums at 3.048 and 3.146 mins.

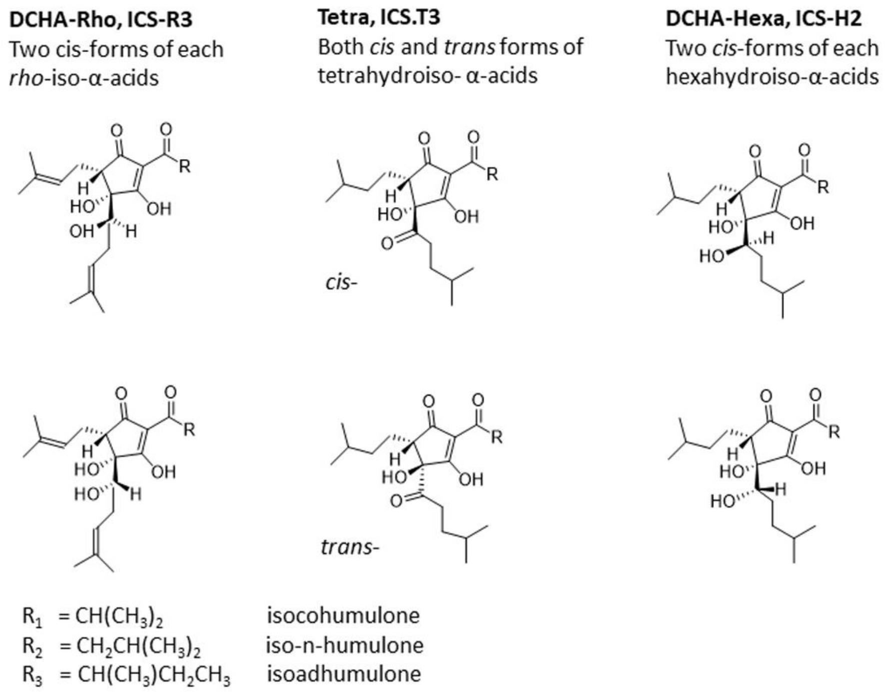

Figure S4. Structures of the reduced iso-α-acids tested.

Table S1 Concentration ranges of iso-α-acids and ethanol in experiments

Stimulants	Range	Supplier	Cell type	Range
Iso-α-acids	0.276 pM - 27.6 µM	Thermo Fisher	SH-SY5Y<br>N2a	0.276 pM - 27.6 µM<br>0.276 nM - 11.0 µM
Iso-α-acids	0.0276 pM - 55.2 µM	Hopsteiner	SH-SY5Y<br>N2a<br>NES	0.0276 pM -55.2 µM<br>0.276 nM - 11.0 µM<br>0.276 pM -55.2 µM
Trans-iso-α-acids	0.276 pM - 55.2 µM	Labor Veritas	SH-SY5Y<br>N2a<br>NES	0.276 pM -55.2µM<br>0.276 nM - 11.0 µM<br>0.276 pM -55.2 µM
Cis-ρ-iso-α-acids	2.76 pM - 55.2 µM	Labor Veritas	SH-SY5Y<br>N2a	2.76 pM - 55.2 µM<br>0.276 nM - 11.0 µM
Hexa-hydro-iso-α-acids	0.276 nM - 11.0 µM	Labor Veritas	SH-SY5Y	0.276 nM - 11.0 µM
Tetra-hydroiso-α-acids	0.276 nM - 11.0 µM	Labor Veritas	SH-SY5Y	0.276 nM - 11.0 µM
Ethanol	0.2 - 75 mM		SH-SY5Y<br>N2a<br>NES	0.2 - 75 mM<br>2 - 20 mM<br>0.2 - 75 mM
